# Matriptase Cleaves EpCAM and TROP2 in Keratinocytes, Destabilizing Both Proteins and Associated Claudins

**DOI:** 10.3390/cells9041027

**Published:** 2020-04-21

**Authors:** Chuan-Jin Wu, Michael Lu, Xu Feng, Gaku Nakato, Mark C. Udey

**Affiliations:** 1 Laboratory of Immune Cell Biology, National Cancer Institute, Bethesda, MD 20892, USA; 2 Experimental Immunology Branch, National Cancer Institute, Bethesda, MD 20892, USA; lumi@mail.nih.gov; 3 Retired from National Cancer Institute, Bethesda, MD 20892, USA; xuf99@yahoo.com; 4 Kanagawa Institute of Industrial Science and Technology, Tonomachi, Kawasaki-ku, Kawasaki-shi, Kanagawa 210-0821, Japan; op-nakato@newkast.or.jp; 5 Dermatology Division, Department of Medicine, Washington University School of Medicine, Saint Louis, MO 63110, USA

**Keywords:** EpCAM, TROP2, matriptase, HAI-1, HAI-2, claudin, keratinocytes, ichthyosis

## Abstract

The homologs EpCAM and TROP2, which both interact with claudin-1 and claudin-7, are frequently coexpressed in epithelia including skin. Intestine uniquely expresses high levels of EpCAM but not TROP2. We previously identified EpCAM as a substrate of the membrane-anchored protease matriptase and linked HAI-2, matriptase, EpCAM and claudin-7 in a pathway that is pivotal for intestinal epithelial cells (IEC) homeostasis. Herein, we reveal that TROP2 is also a matriptase substrate. Matriptase cleaved TROP2 when purified recombinant proteins were mixed in vitro. TROP2, like EpCAM, was also cleaved after co-transfection of matriptase in 293T cells. Neither EpCAM nor TROP2 cleavage was promoted by protease-disabled matriptase or matriptase that harbored the ichthyosis-associated G827R mutation. We confirmed that EpCAM and TROP2 are both expressed in skin and detected cleavage of these proteins in human keratinocytes (HaCaT cells) after the physiologic inhibition of matriptase by HAI proteins was relieved by siRNA knockdown. Knockdown of EpCAM or TROP2 individually had only small effects on claudin-1 and claudin-7 levels, whereas elimination of both markedly diminished claudin levels. HAI-1 knockdown promoted EpCAM and TROP2 cleavage accompanied by reductions in claudins, whereas HAI-2 knockdown had little impact. Double knockdown of HAI-1 and HAI-2 induced nearly complete cleavage of EpCAM and TROP2 and drastic reductions of claudins. These effects were eliminated by concurrent matriptase knockdown. Decreases in claudin levels were also diminished by the lysosomal inhibitor chloroquine and cleaved EpCAM/TROP2 fragments accumulated preferentially. We demonstrate that TROP2 and EpCAM exhibit redundancies with regard to regulation of claudin metabolism and that an HAI, matriptase, EpCAM and claudin pathway analogous to what we described in IECs exists in keratinocytes. This study may offer insights into the mechanistic basis for matriptase dysregulation-induced ichthyosis.

## 1. Introduction

Matriptase (ST14) is a type II membrane serine protease that is expressed in embryonic and all adult epithelia [1]. It is produced as a zymogen and becomes active after endoproteolytic cleavage by prostasin or matriptase itself [1,2,3]. Matriptase regulates epithelial barrier function in vitro and in vivo [4,5] and dysregulation of matriptase causes defects in multiple epithelia. Matriptase-deficiency in mice leads to malformation of stratum corneum and aberrant hair follicle development [6], and matriptase knockout mice die perinatally due to dehydration resulting from severe skin barrier impairment. Mutations in matriptase have been associated with two human syndromes; autosomal recessive ichthyosis with hypotrichosis (ARIH) and ichthyosis, follicular atrophoderma, hypotrichosis, and hypohidrosis (IFAH) [7,8]. Constitutive transgenic expression of matriptase in basal keratinocytes also induces squamous carcinoma in mice [9]. 

Matriptase activity is tightly controlled by its cognate inhibitors HAI (hepatocyte growth factor activator inhibitor)-1 (encoded by *SPINT1*) and HAI-2 (encoded by *SPINT2*) under physiological conditions [10,11]. A mutation-based screen determined that HAI-1 regulates zebrafish skin development and it has been demonstrated that HAI-1 promotes zebrafish skin epithelial integrity via inhibition of matriptase [12]. HAI-1-deficient mice display severe ichthyosis and abnormal hair development [13], and these phenotypes are associated with hyperactivation of matriptase [14]. 

Several matriptase substrates, including urokinase plasminogen activator (uPA), epidermal growth factor receptor (EGFR), and hepatocyte growth factor (HGF), have been identified [4,15]. However, detailed mechanisms by which these proteins contribute to physiological functions of matriptase has often not been elucidated [1,4]. Impaired skin barrier function induced by matriptase mutations has been attributed to defective filaggrin processing [8,16], but intracellular segregation of matriptase and profilaggrin suggests the latter is not a direct matriptase substrate [17]. Changes in profilaggrin processing are also unlikely to explain the matriptase deficiency-promoted hair follicle defect. A report by Chen et al. [18] argued that matriptase regulates keratinocyte proliferation and early differentiation in human skin. Dysregulated intercellular adhesion has been implicated in the impaired skin integrity in zebrafish carrying HAI-1 mutations, but the putative matriptase substrate HGF, a known promoter of epithelial-mesenchymal transitions, does not appear to play a major role [12]. Identification of new matriptase substrates in keratinocytes may facilitate better understanding of matriptase’s role in regulating skin homeostasis. 

We previously reported that EpCAM (also known as CD326 or TROP1) is a physiological substrate of matriptase in intestinal epithelial cells (IEC) [19]. Cleavage of EpCAM by matriptase between Arg80 and Arg81 led to dissociation of EpCAM/claudin-7 complexes and lysosomal degradation of EpCAM and claudin-7 in IECs. The coordinated actions of HAI-2, matriptase, EpCAM and claudin-7 were essential for intestinal epithelial homeostasis [19]. Studies of a mouse model has demonstrated that EpCAM cleavage is enhanced and claudin-7 levels are diminished in intestinal epithelia of *SPINT2* knockout mice which mimic congenital tufting enteropathy [20]. All of these proteins and/or their homologs are also present in skin [6,13,21]. Adult intestinal epithelia is unusual in that it expresses EpCAM but not its homolog TROP2, whereas skin expresses both proteins [21]. It has also been reported that TROP2 interacts with claudin-7 and claudin-1, protecting these claudins from degradation in corneal epithelial cells [21]. Similar to intestinal epithelium, skin constitutes a major barrier that protects the organism from environmental and microbial insults. Herein we report that, like EpCAM, TROP2 is a matriptase substrate. EpCAM and TROP2 had similar roles as regulators of claudins in keratinocytes. We also describe a HAI-1(2)/matriptase/TROP2(1)/claudin cascade that is analogous to the one that we reported in IECs [19]. This work may promote understanding of molecular mechanisms behind physiological and pathological roles of matriptase and HAI-1 in skin.

## 2. Materials and Methods

### 2.1. Antibodies 

Affinity-purified polyclonal rabbit anti-EpCAM antibody (Ab) has been described previously [22]. Monoclonal anti-mouse TROP2 antibody was generated by immunizing rabbits with recombinant protein comprised of the extracellular region of mouse TROP2 fused by human IgG Fc. Polyclonal anti-TROP2, polyclonal goat anti-human HAI-1 (AF1048), sheep anti-matriptase (AF3946), goat anti-mouse HAI-1 (AF1141), and goat anti-mouse HAI-2 (AF1107) Abs were purchased from R & D Systems (Minneapolis, MN, USA). Anti-EpCAM (PA5-19832), anti-claudin-1 (717800), anti-claudin-7 (349100), and anti-occludin (711500) Abs were from Thermo Fisher Scientific (Carlsbad, CA, USA). Rabbit anti-matriptase Ab (IM1014) was from EMD Millipore (Temecula, CA, USA). Polyclonal anti-HAI-2 Ab (HPA011101), mouse anti-Flag mAb (clone M2) and anti-β-actin mAb (clone AC-15) were from Sigma (St. Louis, MO, USA). Anti-E-cadherin mAb was from BD Biosciences (San Jose, CA, USA), and rat anti-HA mAb (clone 3F10) was from Roche (Indianapolis, IN, USA).

### 2.2. Gene Expression Plasmids 

pcDNA3-HAEpCAM has been described [22]. Plasmid expressing Flag-tagged human matriptase was obtained from OriGene (Rockville, MD, USA). PCR-amplified HA-tagged mouse TROP2 cDNA was cloned into pcDNA3. The constructed plasmid was verified by DNA sequencing. Matriptase mutations were generated with a Quickchange Kit (Agilent Technologies, Santa Clara, CA, USA) following the manufacturer’s instructions. 

### 2.3. Cell Culture 

HaCaT cells were purchased from AddexBio (San Diego, CA, USA). Caco-2 cells have been described [22]. The 308 mouse keratinocyte cell line was kindly provided by Dr. Stuart Yuspa (National Cancer Institute, Bethesda, MD, USA). HaCaT cells and 308 cells were grown in DMEM containing 10% fetal bovine serum (FBS). Caco-2 cells were grown in DMEM supplemented with 10% FBS, 15 mM HEPES (pH 7.4) and non-essential amino acids. 

### 2.4. Treatment of TROP2 with Recombinant Matriptase In Vitro 

Catalytically active recombinant mouse matriptase was purchased from R&D Systems. Recombinant mouse TROP2-hIgG protein was affinity-purified using protein A-sepharose (GE Healthcare, Pittsburgh, PA, USA) from media of cultured 293F cells transfected with a plasmid encoding the mouse TROP2 extracellular domain fused to human IgG1 Fc fragment using Turbofect (Thermo Fisher Scientific). For the in vitro cleavage assay, recombinant TROP2 was mixed with recombinant matriptase in 100 μL reaction buffer (50 mM Tris, pH 8.5, 100 mM NaCl) and incubated at 37 °C for 1 h. 

### 2.5. Transfection of 293 T Cells for Protein Expression 

Empty vectors or vectors encoding HA-tagged EpCAM, HA-tagged TROP2, or Flag-tagged matriptase were transfected into 293 T cells with Fugene 6 (Promega, Madison, WI, USA) following the manufacturer’s instructions. 

### 2.6. Knockdown of Protein Expression by siRNA Transfection 

Matriptase siRNAs (HSS110268) were purchased from Thermo Fisher Scientific. Two human EpCAM siRNAs and human SPINT2 siRNA have been described previously [19,22]. Human TROP2 and human SPINT1 siRNAs and mouse SPINT2 siRNAs were from Sigma. Negative control siRNA was purchased from Sigma or Qiagen. HaCaT cells and 308 cells were transfected with negative control siRNA, EpCAM siRNA, TROP2 siRNA, SPINT1 siRNA, SPINT2 siRNA or matriptase siRNA duplexes using electroporation (Gene pulser, Bio-Rad Laboratories, Hercules, CA, USA) at 300 V for 20 s. Transfected cells were harvested and lysed 72 h after electroporation. Protein levels and/or protein sizes were determined by Western blotting. 

### 2.7. Gel Electrophoresis and Immunoblotting 

Cells were lysed with RIPA lysis buffer and protein concentrations were determined using a Bradford protein assay (Bio-Rad Laboratories, Hercules, CA, USA) [22]. Cell lysates were cleared by centrifugation and resolved by SDS-PAGE (Nupage Bis-Tris gels, Thermo Fisher Scientific) and proteins were visualized via Coomassie blue staining or immunoblotting. For immunoblotting, proteins were transferred onto nitrocellulose membranes after SDS-PAGE separation and membranes were subsequently incubated with the indicated Abs. Immunoreactive proteins on the blotted membrane were visualized using horseradish peroxidase-conjugated secondary Ab (Jackson ImmunoResearch, West Grove, PA, USA) and enhanced chemiluminescence (Thermo Fisher Scientific). Protein band intensities were quantified using densitometry and NIH ImageJ software.

### 2.8. Mouse Study 

C56BL/6 mice were obtained from Charles River Laboratories (Frederick, MD, USA) and housed in a pathogen-free American Association for Laboratory Animal Care (AALAC)-accredited facility on the NIH campus in Bethesda, MD, USA. Animal studies were carried out following guidelines established by Research Animal Resource Center, NIH under the auspices of an animal study protocol that had been approved by the National Cancer Institute Animal Care and Use Committee (ACUC). 

### 2.9. Statistics 

Probability (p) values for multiple group comparisons were calculated using two-way ANOVA. P values for two group comparison were calculated using the Student’s t test. Aggregate results of multiple experiments are displayed as means ± SEM. 

## 3. Results 

### 3.1. Differential Expression of HAI-1, HAI-2, EpCAM, and TROP2 in Skin and Intestine

**Figure 1:**
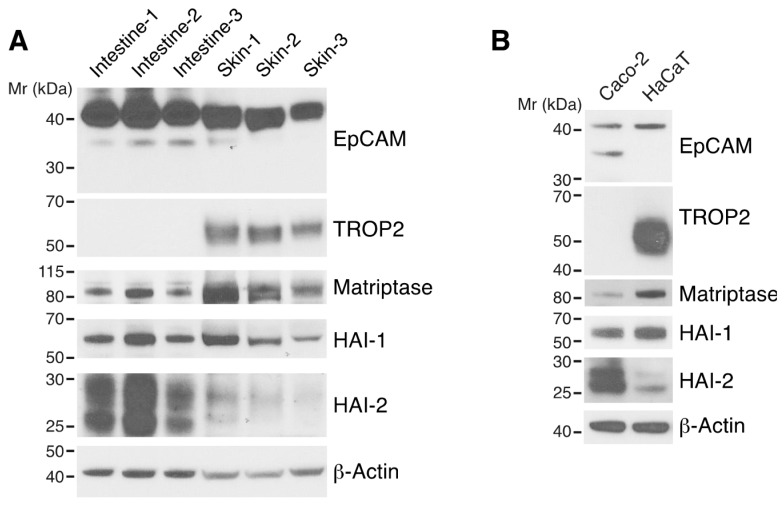
Expression of HAI-1, HAI-2, matriptase, EpCAM, and TROP2 in skin and intestine. RIPA lysates of trunk skin and small intestine from 8–10 week-old C57BL/6 mice (**A**) and Caco-2 intestinal epithelial cells (IECs) and HaCaT keratinocytes (**B**) were resolved via reduced SDS-PAGE and immunoblotted with anti-HAI-1, anti-HAI-2, anti-matriptase, anti-EpCAM, anti-TROP2. β-actin was used as a loading control. Representative data from 1 of 3 experiments for (**B**) is shown.

We were interested in determining if the functional HAI/matriptase/EpCAM/claudin pathway that we had identified in intestine [19] also exists in skin. We first analyzed expression of the major components and their homologs in the pathway in mouse trunk skin using Western blotting. HAI-1, HAI-2, matriptase and EpCAM were all detected in skin. In contrast to small intestine, skin expressed not only EpCAM but also TROP2 (Figure 1A). Markedly less HAI-2 was expressed in skin as compared with intestine, and the relative ratios of HAI-2/HAI-1 were more than 8 times less in skin than intestine (Figure 1A and Figure S1). A similar pattern of expression of the related proteins was observed in well characterized intestinal epithelial and keratinocyte cell lines (Caco2 and HaCaT cells, respectively; Figure 1B). 

### 3.2. Both EpCAM and TROP2 Modulate Associated Claudins in Keratinocytes

**Figure 2:**
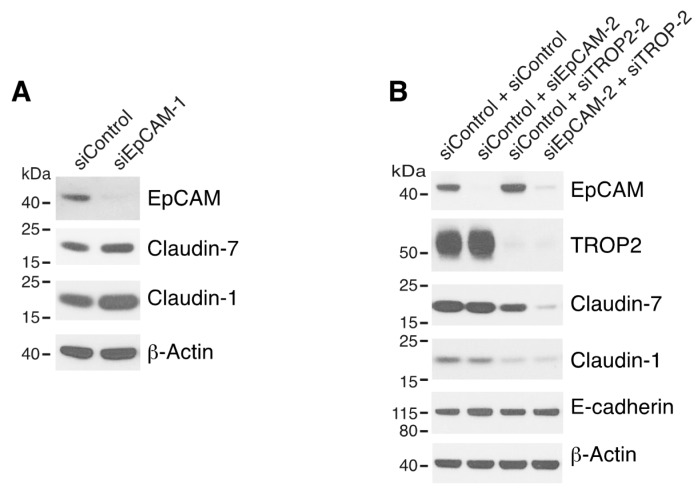
Both EpCAM and TROP2 regulate claudin expression in keratinocytes. HaCaT cells were transfected with control or EpCAM siRNAs (**A**), control, EpCAM, TROP2, or EpCAM and TROP2 siRNAs (**B**) using electroporation. 72 h after transfection, cell lysates were prepared, resolved using SDS-PAGE and immunoblotted with anti-EpCAM, anti-TROP2, anti-claudin-7 or anti-claudin-1 to assess corresponding protein levels. β-actin was used as a loading control.

It has been reported that TROP2, like EpCAM, interacts with claudin-1 and caludin-7 and stabilizes these claudins in corneal epithelial cells [21,22]. To determine if EpCAM and/or TROP2 influence claudin-1 and/or claudin-7 in keratinocytes, we analyzed claudin expression after knocking down EpCAM and/or TROP2 via siRNA transfection. In contrast to what we previously observed in Caco-2 and T84 IEC cells [22], efficient downregulation of EpCAM expression alone did not reduce claudin 1/7 levels in HaCaT cells (Figure 2A and Figure S2). We then proceeded to evaluate the impacts on claudins by inhibition of both EpCAM and TROP2. To minimize the chance that the observed impacts might be due to siRNA off-target effects, two or more different EpCAM and TROP2 siRNAs were used in these experiments. Modest decreases in claudin-1 and claudin-7 levels was observed in TROP2 siRNA transfected cells (Figure 2B). However, levels of both claudins (but not E-cadherin) were dramatically diminished by simultaneous knockdown of EpCAM and TROP2 (Figure 2B and Figure S2). It appears that reductions in claudin levels correlated with the knockdown efficacies of different EpCAM and TROP2 siRNA combinations (Figure S2A). These results demonstrated that EpCAM and TROP2 coordinately regulate selected claudins in keratinocytes. 

### 3.3. Matriptase Cleaves TROP2 in vitro and Co-Expressed Matriptase Cleaves TROP2 in Cells.

**Figure 3:**
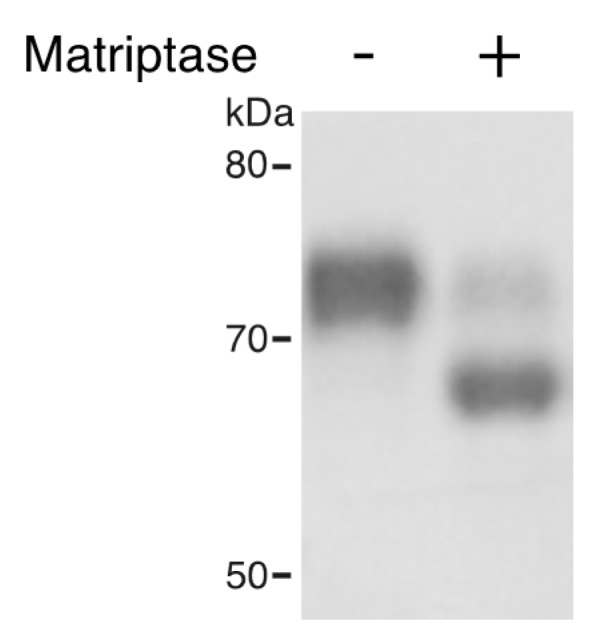
Matriptase cleaves TROP2 in vitro. 4 μg of recombinant TROP2-Ig was incubated with or without 0.04 μg of recombinant matriptase in 100 μL of reaction buffer at 37 °C for 1 h. Reaction products were resolved via SDS-PAGE and stained with Coomassie blue. Representative data from 1 of 3 experiments is shown.

**Figure 4:**
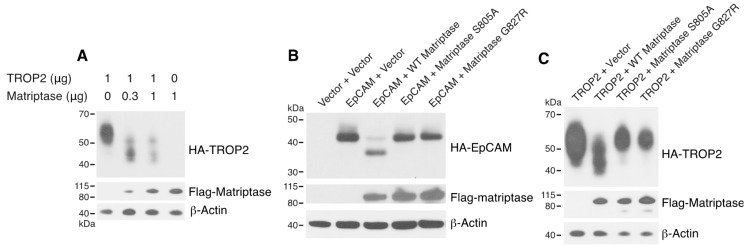
Active matriptase, but not inactive S805A or disease-associated G827R mutant matriptase, promotes cleavage of EpCAM and TROP2. 293T cells were transfected using Fugene 6 with fixed amounts of pcDNA3 encoding HA-TROP2 and varying amounts of Flag-matriptase expression plasmid (**A**), pcDNA3 encoding HA-EpCAM and plasmid encoding Flag-matriptase, Flag-matriptase S805A, or Flag-matriptase G827R (**B**), pcDNA3 encoding HA-TROP2 and plasmids encoding Flag-matriptase or mutants (**C**). 48 h after transfection cell lysates were prepared and resolved, and the indicated epitope-tagged proteins were analyzed via Western blotting using anti-HA and anti-Flag. Representative data from 1 of 3 experiments is shown.

Human TROP2 shares 48% amino acid identity and 62% amino acid similarity with human EpCAM. In addition, a homologous arginine that we identified as the matriptase cleavage site for EpCAM (Arg80) is present in human TROP2 (Arg87) and murine TROP2 (Arg81) (Figure S3). To determine if matriptase cleaves TROP2 directly, we incubated recombinant mTROP2-IgG with recombinant active matriptase. Mouse matriptase efficiently cleaved TROP2 and the cleavage resulted in a ~6 kDa reduction in the molecular size of mouse TROP2-IgG (Figure 3), consistent with cleavage at the site Arg81, which is homologous to the EpCAM cleavage site. Furthermore, co-expressed matriptase led to cleavage of TROP2 in 293T cells in a dose-dependent manner (Figure 4A). These results demonstrated that like EpCAM, TROP2 is cleaved by matriptase in vitro and suggest that analogous cleavage also occurs in cells and presumably in vivo. EpCAM (Figure 4B) and TROP2 (Figure 4C) cleavage was not promoted by the protease-inactive matriptase S805A mutant [23], indicating that matriptase protease activity is required for EpCAM and TROP2 cleavage in cells. Matriptase G827R mutation has previously been identified in some ARIH patients [7]. Interestingly, the ARIH-associated G827R mutation also prevented matriptase cleavage of EpCAM (Figure 4B) and TROP2 (Figure 4C). 

### 3.4. HAI-1 and HAI-2 Attenuate EpCAM and TROP2 Cleavage and Protect Claudins in Keratinocytes

**Figure 5:**
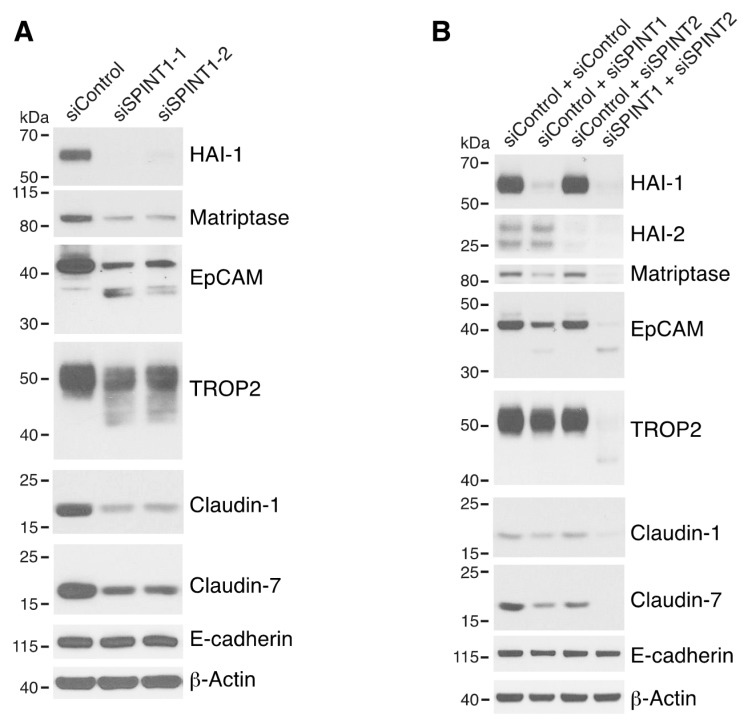
HAI-1 and HAI-2 regulate EpCAM and TROP2 cleavage and claudin levels. HaCaT cells were transfected using electroporation with control siRNA or one of two different SPINT1 siRNAs (siSPINT1-1 and siSPINT1-2) (**A**), control, SPINT1, SPINT2, or SPINT1, and SPINT2 siRNAs (**B**). After 72 h, cell lysates were prepared, subjected to electrophoresis and analyzed via Western blotting for HA1-1, HAI-2, matriptase, EpCAM, TROP2, claudin-1, claudin-7 and E-cadherin. β-actin was used as a loading control. Representative data from 1 of 3–4 experiments is shown.

We next determined if matriptase-mediated proteolysis of endogenous EpCAM and TROP2 occurred in HaCaT cells. Despite observation of little cleavage of EpCAM and TROP2 in untransfected (Figure 1B) and control siRNA transfected HaCaT cells (Figure 5A), EpCAM cleavage and TROP2 cleavage were readily apparent in HAI-1 knockdown cells (Figure 5A). This was accompanied by claudin-1 and claudin-7 downregulation (Figure 5A). Small amounts of EpCAM cleavage and TROP2 cleavage were also observed in mouse 308 keratinocytes (Figure S4). In striking contrast to our previous observations of dramatic increases in EpCAM cleavage and reductions in claudin-7 levels in HAI-2 knockdown IEC cells [19], knockdown of HAI-2 in keratinocytes did not induce EpCAM/TROP2 cleavage or reduce claudin levels (HaCaT cell, Figure 5B and Figure S5), or slightly increased EpCAM/TROP2 cleavage (308 cells, Figure S4). These results are consistent with notion that HAI-1 plays a more prominent role than HAI-2 in inhibiting matriptase and in regulating matriptase function in skin than in intestine [13,14,17]. Interestingly, simultaneous inhibition of both HAI-1 and HAI-2 led to marked increases in EpCAM and TROP2 cleavage and striking downregulation of claudin-1 and claudin-7 (Figure 5B and Figure S5), implying that HAI-1 and HAI-2 are redundant or cooperative in inhibiting matriptase action on endogenous substrates in keratinocytes. Again, the 6–10 kD molecular size reduction (Figure 4A,C and Figure 5A,B) in TROP2 after cleavage suggested that matriptase cleaves hTROP2 at the homologous EpCAM cleavage site in TROP2, Arg87 (Figure S3). However, it is possible that matriptase cleaves TROP2 at one or two additional sites that are close to Arg87, since multiple cleavage products were seen in several experiments (Figure 4A,C and Figure 5A,B). Potentially relevant to this, there are at least two lysine/arginine’s (Lys84 and Arg92) that are located in close proximity to Arg87 in hTROP2 (Figure S3).

### 3.5. Matriptase Cleaves EpCAM and TROP2 to Induce Degradation of EpCAM, TROP2 and Claudins in Lysosomes in Keratinocytes 

**Figure 6:**
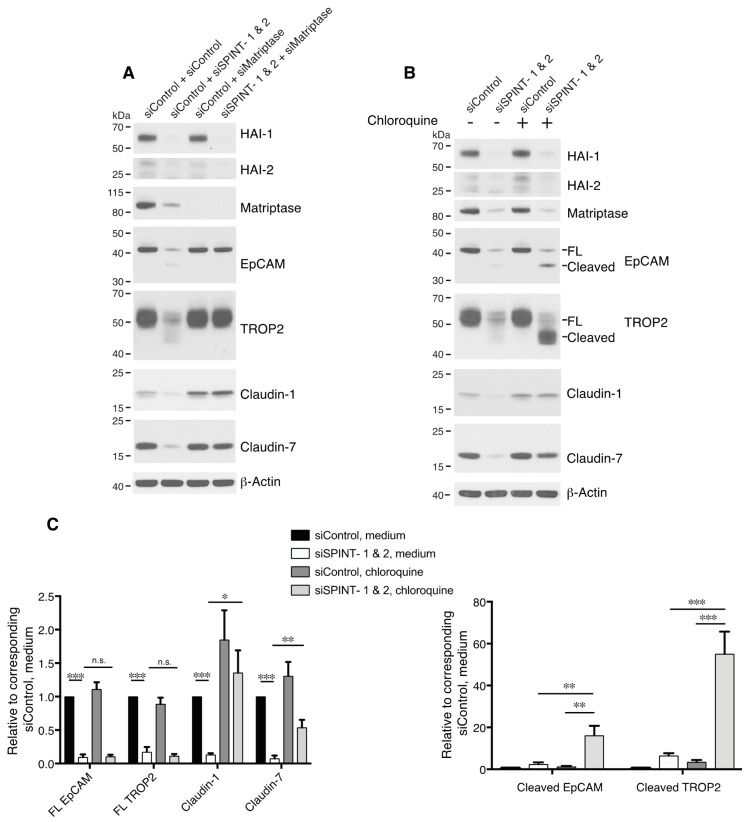
HAIs act via matriptase to regulate degradation of EpCAM, TROP2, and claudins in lysosomes. HaCaT cells were transfected using electroporation with control or SPINT1 plus SPINT2, matriptase, or SPINT1, SPINT2 plus matriptase siRNAs (**A**), or control or SPINT1 and SPINT2 siRNAs (**B**). Before being harvested at 72 h after transfection (**A**,**B**), cells were treated with or without 100 μM chloroquine for 20 h (**B**). RIPA cell lysates were resolved using SDS-PAGE and immunoblotted with anti-HAI-1, anti-HAI-2, anti-matriptase, anti-EpCAM anti-TROP2, anti-claudin-1, or anti-claudin-7. β-actin was used as a loading control. Representative data from 1 of 5 experiments is shown. (**C**) Band intensities corresponding to full-length (FL) EpCAM, FL TROP2, claudin-1 and claudin-7 (left panel) and cleaved EpCAM and cleaved TROP2 (right panel) were quantified and normalized to β-actin. Data are plotted as ratios (means ± SEM) relative to corresponding untreated siControls (*n* = 5). A two-way ANOVA was used to calculate p values for multiple group comparisons to assess mean differences between groups (* *p* < 0.05, ** *p* < 0.01, *** *p* < 0.001 or 0.0001, n.s. *p* > 0.05).

To address the possibility that HAI-1 and HAI-2 were acting via inhibition of serine proteases other than matriptase [17,24], we analyzed EpCAM and TROP2 cleavage in HaCaT cells after transfecting matriptase siRNA along with SPINT1 and SPINT2 siRNAs (Figure 6A). Knockdown of matriptase eliminated HAI-1/2 inhibition-induced EpCAM and TROP2 cleavage and claudin downregulation (Figure 6A), suggesting that HAI-1/2 regulation of EpCAM/TROP2 and claudins in keratinocytes is via inhibition of matriptase. These results also revealed that, like EpCAM, TROP2 is an intrinsic matriptase substrate in cells. Marked decreases in total EpCAM and TROP2 (but not E-cadherin) levels were observed in the HaCaT cells that were transfected with SPINT1 and SPINT2 siRNAs (Figure 5B and Figure 6A). We previously demonstrated that matripase-cleaved EpCAM and claudins disassociate, internalize and are degraded in lysosomes [19,22], and it was reported that TROP2 knockdown-induced claudin reductions are not transcription-related [21]. To determine whether the loss of EpCAM/TROP2 and/or claudin is caused by protein degradation in lysosomes, we treated SPINT1/2 siRNA-transfected cells with chloroquine. This lysosomal inhibitor largely rescued claudin-1 and claudin-7 losses resulting from HAI-1 and HAI-2 inhibition (Figure 6B,C). In addition, chloroquine increased accumulation of cleaved EpCAM and cleaved TROP2, but not full-length forms of these proteins (Figure 6B,C). These results demonstrated that matriptase-cleaved EpCAM and TROP2 proteins are unstable in keratinocytes, that they are degraded in lysosomes, and that they lose their ability to stabilize claudins.

## 4. Discussion

In this study, we confirmed that EpCAM and TROP2 are present in keratinocytes, and we demonstrated that both EpCAM and TROP2 regulate the levels of claudin-1 and claudin-7 in these cells. TROP2 was cleaved by matriptase in vitro and TROP2 cleavage was promoted by matriptase overexpression in cells, indicating that, like EpCAM, TROP2 is a substrate of matriptase. Matriptase cleavage induced lysosomal degradation of EpCAM/TROP2 and associated claudins. Matriptase-mediated cleavage of EpCAM and TROP2 and downregulation of claudins was inhibited by HAI-1 and HAI-2 in keratinocytes. Thus, analogous HAI/matriptase/EpCAM/claudin pathways exist in keratinocytes and IECs. The pathways in each tissue have distinct features, however. In keratinocytes, both EpCAM and TROP2 are present, and both regulate claudins. HAI-2 is the dominant HAI regulator of matriptase-mediated cleavage of EpCAM in IECs [19,20], whereas HAI-1 plays a more important role than HAI-2 in regulating matriptase cleavage of EpCAM/TROP2 in keratinocytes. Claudin-7 is the major claudin that is modulated by matriptase and HAIs in IECs [11,19]. In keratinocytes, claudin-1 is also regulated by these proteins. 

Patients with EpCAM mutations and EpCAM knockout mice display severe intestinal dysfunction, but they do not exhibit obvious extra-intestinal abnormalities [25,26,27]. One possible explanation is that EpCAM is strongly expressed in the intestine while TROP2 is virtually absent. In most other epithelia, both EpCAM and TROP2 are present, and in these epithelia one protein may compensate for the other. TROP2 is highly homologous to EpCAM, and both TROP2 and EpCAM interact with claudin-7 and claudin-1 [21,22]. The present study demonstrates that both EpCAM and TROP2 regulate claudins in keratinocytes. We have determined that transgenic expression of TROP2 in murine intestine was able to at least partially reverse intestinal dysfunction in EpCAM KO mice, enabling survival (Nakato G and Udey MC, unpublished data). TROP2’s ability to interact with and stabilize claudins may explain this observation. It is possible that TROP2 and EpCAM could also functionally compensate for each other in skin.

We have also shown that both siRNA inhibition of EpCAM and TROP2 expression and matriptase cleavage of EpCAM and TROP2 led to downregulation of claudin-1 and claudin-7 in keratinocytes. It is unknown whether or not claudin-7 plays an important role in skin biology, but studies of knockout mice demonstrated that claudin-1 is critical for skin barrier function. Claudin-1 knockout mice die within 1–2 days after birth because of dehydration caused by skin barrier dysfunction [28]. We observed that inhibition of HAI-1 in HaCaT cells led to activation of matriptase and decreases in claudin-1 and claudin-7 levels. We propose that alterations in claudin-1 stability contributes to, or is responsible for, HAI-1 deficiency-induced ichthyosis. 

EpCAM and TROP2 constitutively interact with claudin-1 and claudin-7 [21,22,29]. We previously showed that matriptase co-localizes with EpCAM in intestinal epithelia and that matriptase interacts with EpCAM in intestinal epithelial cells [19]. Protein structural studies have demonstrated that both EpCAM and TROP2 form homodimers [30,31]. Also, it has been reported claudins may homo- or hetero-oligomerize [32,33] and that claudin-1 and claudin-7 interact [22,34]. This suggests that EpCAM and TROP2 could be present in complexes with the potential to interact with, and be regulated by, matriptase. It is not known whether or not EpCAM and TROP2 heterodimerize and if matriptase interacts with EpCAM and/or TROP2 directly in skin cells. More details regarding matriptase/EpCAM/TROP2/claudin complex formation and complex regulation by other proteins such as HAI-1/2 await further studies.

In contrast to what we observed in the IEC cell line Caco2 [19], HAI-2 inhibition by siRNA in keratinocytes minimally increased EpCAM and TROP2 cleavage and claudin loss. Two recent studies demonstrated that EpCAM was robustly cleaved and claudin-7 was markedly downregulated in the intestinal epithelia of *SPINT2* KO mice [20,35]. The reasons for the discrepancy regarding HAI-2’s role in inhibiting matriptase cleavage of TROP proteins in IECs and in keratinocytes are unknown. Marked differences in HAI-1/HAI-2 protein level ratios in skin and intestine (Figure 1A and Figure S1) may be relevant. Our siRNA transfection experiment suggested that HAI-1 plays a more prominent role in inhibiting matriptase in keratinocytes than HAI-2 does. These results are consistent with the concept that HAI-2 is more important than HAI-1 in intestine, and that the reverse is true in skin [11,13,20,36]. We detected much more EpCAM and TROP2 cleavage and claudin loss in double knockdown cells than in single SPINT1 siRNA- or SPINT2 siRNA- transfected cells. These results indicate that HAI-1 and HAI-2 may play a redundant or cooperating role in inhibiting matriptase in keratinocytes. While it is known that both HAI-1 and HAI-2 inhibit matriptase activity, the precise roles of these homologous proteins in regulating matriptase are currently unclear. Identification of EpCAM and TROP2 as physiologically relevant substrates of matriptase may create opportunities to study mechanistic aspects of matriptase regulation in detail. Understanding the molecular mechanisms for activation and inhibition of matriptase is key to designing strategies to treat matriptase dysregulation-induced conditions. 

Finally, it is not clear why mutations in matriptase cause ichthyosis. Studies using synthetic peptide substrates suggested that the G827R mutation inactivates protease activity [23,37]. Consistent with this, in this study we showed that the matriptase G827R mutant also fails to promote cleavage of the physiological substrates EpCAM and TROP2. Additional studies are needed to further evaluate the functional importance of the HAI/matriptase/TROP/claudin pathway in skin and to determine if some rare, or perhaps common, skin diseases are a consequence of dysregulation of this pathway. EpCAM, TROP2, claudins, matriptase, and HAI proteins have all been reported roles in cancer [38,39,40,41,42,43,44,45]. Our demonstration of the physiological relationship among these proteins may offer clues for a better understanding of their roles in cancer at molecular level.

## Supplementary Materials

The following are available online at https://www.mdpi.com/2073-4409/9/4/1027/s1, Figure S1: Differential HAI-1 and HAI-2 expression in mouse intestine and skin, Figure S2: EpCAM and TROP2 regulate keratinocyte claudin levels, Figure S3: Protein homology of human EpCAM and human TROP2, Figure S4: Cleavages of EpCAM and TROP2 in mouse keratinocytes, Figure S5: A replicate experiment of Figure 5B.
