# Supplementary material for: Matriptase Cleaves EpCAM and TROP2 in Keratinocytes, Destabilizing Both Proteins and Associated Claudins
Source: Cells. 2020 Apr 21;9(4):1027. doi: 10.3390/cells9041027 (PMC7226414; doi:10.3390/cells9041027)
Supplement: Supplementary file 1 [file cells-09-01027-s001.pdf]

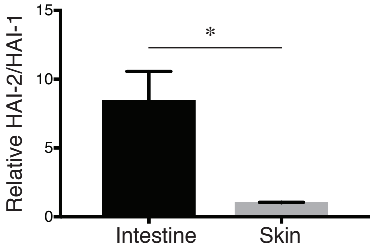

Supplemental Figure 1. Differential HAI-1 and HAI-2 expression in mouse intestine and skin. Band intensities of HAI-1 and HAI-2 in Fig. 1A were quantified. Relative ratios of intestine or skin HAI-2 and HAI-1 were calculated for each animal and are depicted (means ± SEM). The Student t test was used to calculate p values (*$p<0.05$).

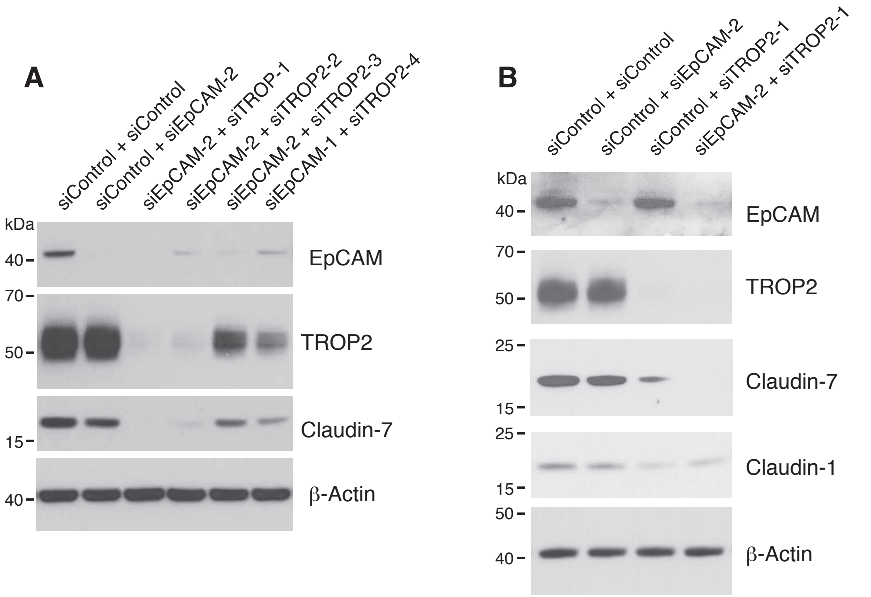

Supplemental Figure 2. EpCAM and TROP2 regulate keratinocyte claudin levels. HaCaT cells were transfected with combinations of two siRNAs of control siRNA, two different EpCAM siRNAs and 4 different TROP2 siRNAs as indicated (**A**), control siRNA, EpCAM siRNA (siEpCAM-2), TROP2 siRNA (siTROP-1), or EpCAM and TROP2 siRNAs (**B**) using electroporation. Three days later, cell lysates were prepared and resolved using SDS-PAGE. Western blotting was conducted to determine corresponding protein levels by probing with anti-EpCAM, anti-TROP2, anti-claudin-7 or anti-claudin-1 Ab. β-actin was used as a loading control.

EpCAM vs. TROP2

Identities = 156 (48%)   Similarities = 62 (19%)

```
EpCAM    1        MAPPQVLAFGLLLAAATATFAAAQEECVCENYKLAVNCFVNNNRQCQCTSVGA  53
TROP2    1 MARGPGLAPPPLRLPLLLLVLAAVTGHTAAQDNCTCPTNKMTVCSPDGPGGRCQCRALGS  60
                    **  *   **. ** .  .***. * * . *..* .  . . .*** ..*.
                                     ■
EpCAM   54 QNTVICSKLAAKCLVMKAEMNGSKLGR-RAKP-EGALQNNDGLYDPDCDESGLFKAKQCN 111
TROP2   61 GMAVDCSTLTSKCLLLKARMSAPKNARTLVRPSEHALVDNDGLYDPDCDPEGRFKARQCN 120
             .* ** *..***..** *   *  *   .* * **  **********  * ***.***

EpCAM  112 GTSTCWCVNTAGVRRTDK-DTEITCSERVRTYWIIIELKHKAREKPYDSKSLRTALQKEI 170
TROP2  121 QTSVCWCVNSVGVRRTDKGDLSLRCDELVRTHHILIDLRHRPTAGAFNHSDLDAELRRLF 180
            ** *****. ******* *  . * * ***  *.*.*.*.     .    * . *..

EpCAM  171 TTRYQLDPKFITSILYENNVITIDLVQNSSQKTQNDVDIADVAYYFEKDVKGESLFHSKK 230
TROP2  181 RERYRLHPKFVAAVHYEQPTIQIELRQNTSQKAAGDVDIGDAAYYFERDIKGESLFQGRG 240
             **.* ***.... **.  * *.* **.***. .**** * *****.*.******. .

EpCAM  231 -MDLTVNGEQLDLDPGQTLIYYVDEKAPEFSMQGLKAGVIAVIVVVVIAVVAGIVVLVIS 289
TROP2  241 GLDLRVRGEPLQVER--TLIYYLDEIPPKFSMKRLTAGLIAVIVVVVVALVAGMAVLVIT 298
            .** * ** *  ..   *****.**  * ***. * **.********.*.***. ****.

EpCAM  290 RKKRMAKYEKAEIKEMGEMHRELNA 314
TROP2  299 NRRKSGKYKKVEIKELGELRKEPSL 323
            ...  ** * ****.**...*
```

Supplemental Figure 3. Protein homology of human EpCAM and human TROP2. Protein sequence homology between human EpCAM (accession number: NP_002345) and human TROP2 (accession number: NP_002344) was calculated using the program ClustaIW. * identical amino acid, **.** similar amino acids, ■ labels the homologous arginine after which EpCAM is cleaved by matriptase.

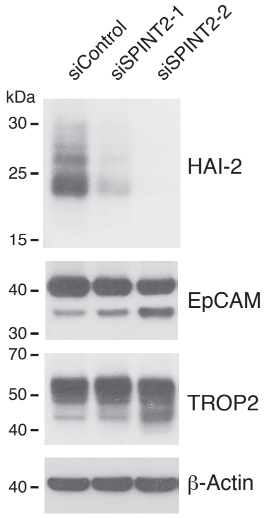

Supplemental Figure 4. Cleavages of EpCAM and TROP2 in mouse keratinocytes. 308 cells were transfected with control siRNA or one of two different mouse SPINT2 siRNAs with electroporation. Cells were harvested and lysed on day 4 after transfection. Cell lysates were resolved with SDS-PAGE and blotted with anti-HAI-2, anti-EpCAM or anti-TROP2 Ab.

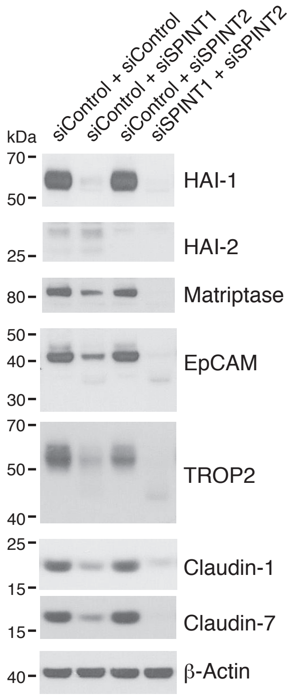

Supplemental Figure 5. A replicate experiment of Figure 5B. HaCaT cells were transfected using electroporation with control, SPINT1, SPINT2, or SPINT1 and SPINT2 siRNAs. After 72 h, cells were harvested and lysed. Cell lysates were resolved by electrophoresis and determined via Western blotting for HA1-1, HAI-2, matriptase, EpCAM, TROP2, claudin-1, claudin-7 and β-actin.
